# Sex-based differences in developing heart failure after ST-segment elevation myocardial infarction

**DOI:** 10.3389/fcvm.2026.1906470

**Published:** 2026-08-26

**Authors:** Yuetao Xie, Wenjing Wang, Xuelian Song, Man Gao, Feifei Zhang, Yingxiao Li, Jiaqi Wang, Yi Dang, Xiaoyong Qi

**Affiliations:** 1 Department of Cardiology, Hebei General Hospital, Shijiazhuang, Hebei, China; 2 Hebei Key Laboratory of Precision Medicine Translational Research on Cardiovascular Diseases, Hebei General Hospital, Shijiazhuang, Hebei, China; 3 Shijiazhuang Cardiovascular Disease Clinical Medical Research Center, Hebei General Hospital, Shijiazhuang, Hebei, China

**Keywords:** heart failure, percutaneous coronary intervention, sex differences, ST-segment elevation myocardial infarction, women

## Abstract

**Background:**

Previous studies have shown that although advances in primary percutaneous coronary intervention (PCI) and medical therapy have significantly improved the survival of patients with ST-segment elevation myocardial infarction (STEMI), the long-term burden of post-infarction heart failure (HF) remains. Studies on sex differences in the occurrence of HF after STEMI are limited. The aim of this study is to investigate the risk factors and gender differences of HF in STEMI patients after PCI.

**Methods:**

We conducted a retrospective cohort study, including a total of 1,040 consecutive patients with STEMI. Clinical, laboratory and surgical-related data of the patients were collected. The primary endpoint was the incidence of new-onset HF, and the secondary endpoint was all-cause mortality during the follow-up period. The cumulative event survival rate was estimated using the Kaplan–Meier method. Univariate and multivariate Cox proportional hazards regression analyses were used to determine the independent predictors of HF. Through subgroup and interaction analyses, the consistency of the association between gender and heart failure in different clinical subgroups was evaluated.

**Results:**

Compared to men, women were significantly older, had a higher prevalence of diabetes, and experienced longer door-to-balloon times. During follow-up, women exhibited a significantly higher crude incidence of HF (25.88% *vs*. 17.86%, *P* = 0.007) and all-cause mortality (12.72% *vs*. 6.53%, *P* = 0.002). Kaplan–Meier analysis confirmed a higher risk of HF in women (Log-rank *P* = 0.006). In univariate Cox analysis, female sex was associated with an increased risk of HF. However, after conducting multivariate adjustments, women no longer served as an independent predictor for HF. Significant interactions were observed between sex and diabetes mellitus (*P _interaction_* = 0.010) and Killip class (*P _interaction_* < 0.001). Women with diabetes, a longer time from symptom onset to first medical contact, and a Killip class of II or above have a much higher risk of developing heart failure. Mediation analysis indicated that diabetes mellitus acted as a significant mediator, explaining 21.76% of the total effect of sex on new-onset HF.

**Conclusion:**

Women have higher HF morbidity and mortality after STEMI, but this difference is mainly driven by their older age and greater burden of comorbidities, not just biological sex.

## Introduction

ST-segment elevation myocardial infarction (STEMI) remains a leading cause of morbidity and mortality worldwide, and although advances in primary percutaneous coronary intervention (PCI) and medical therapy have significantly improved survival, the long-term burden of post-infarction heart failure (HF) remains (1–3). HF has a prevalence of 1%–2% in the adult population and more than 10% in people older than 70 years in developed countries (4, 5). In addition, studies have reported rates of post-STEMI heart failure ranging from 4% to 28% (6, 7). Previous observational findings underscore that patients suffering from heart failure triggered by acute coronary syndrome (ACS) face a significantly poorer survival outlook compared to ACS patients who do not progress to heart failure (8). The onset of HF after STEMI not only seriously impairs the function and quality of life of patients but also is an effective predictor of long-term mortality, which brings a huge economic burden to society and families.

In recent years, the evolutionary understanding of acute coronary syndrome (ACS) mechanisms has underscored emerging clinical opportunities for targeted management (9). However, translating these pathophysiological insights into equitable sex-specific outcomes remains a challenge. Accumulating evidence indicates that female patients frequently present with atypical symptoms, such as dyspnea, fatigue, or epigastric discomfort, rather than classic chest pain. As validated by a landmark systematic review and meta-analysis, these subtle clinical presentations substantially contribute to prolonged pre-hospital and diagnostic delays in women, which may inadvertently extend total ischemic time and exacerbate subsequent adverse cardiac remodeling (10). Few studies have investigated sex differences in the occurrence of HF after STEMI (11, 12). Several reports suggest that women are at higher risk for acute heart failure and death in STEMI compared with men (13). Women with STEMI who are in Killip class at admission are at higher risk for in-hospital HF (14). Therefore, our study aimed to evaluate sex differences in the development of HF and its impact on all-cause mortality in STEMI patients treated with PCI.

## Methods

### Study design and population

We conducted a retrospective single-center study of consecutive STEMI patients who underwent primary PCI at Hebei General Hospital from June 2017 to December 2020. This study has been approved by the Ethics Committee of Hebei General Hospital. This study strictly follows the Declaration of Helsinki.

STEMI was defined according to the Fourth Universal Definition of Myocardial Infarction (15). The treatment approach of all patients followed the recommendations of the European Society of Cardiology guidelines (16). Exclusion criteria included 1) rescue PCI after fibrinolytic therapy; 2) prior diagnosis of heart failure; 3) Cancer; 4) prior coronary artery bypass grafting surgery; 5) prior PCI/PTCA; 6) death during hospitalization; 7) lack of relevant data; 8) were lost to follow-up after discharge. Data on all demographic, clinical, laboratory, and echocardiographic parameters were collected by review of electronic medical records. Hypertension was defined as blood pressure >140/90mmHg, or treatment with antihypertensive drugs. In addition, DM was defined as fasting blood glucose > 126 mg/dL or being treated with oral antidiabetic drugs or insulin.

#### Data collection and definitions

Following the diagnosis of STEMI, patients received a loading dose of clopidogrel (300 mg) or ticagrelor (180 mg) plus aspirin (300 mg). Unfractionated heparin (100 μg/kg) was administered intravenously before coronary intervention. Coronary angiography was performed using the Judkins technique, primarily through the radial approach. TIMI flow grades were independently evaluated by three interventional cardiologists blinded to the patients’ clinical characteristics. No-reflow phenomenon (NRP) is defined as TIMI blood flow grade≤2, and no dissection, distal flushing, spasm, etc., occurred during angiography (17). Medications, including angiotensin-converting enzyme inhibitors, beta-blockers, and statins, were prescribed by the treating cardiologist. Data on family history of smoking, hypertension, coronary heart disease, hyperlipidemia, and prior medication use were obtained by patient self-report. Left ventricular ejection fraction (LVEF) was assessed by experienced echocardiographers using Simpson's method via transthoracic echocardiography.

#### Follow-up and study endpoints

Follow-up data were collected through medical records, outpatient assessments, and telephone interviews. Patients who failed to attend regular follow-up visits were contacted by telephone and asked to provide their medical records from other hospitals to determine whether they had reached the endpoint. The study's primary end point was the first occurrence of new-onset HF after discharge, and the average follow-up time was 42.31 months. New-onset HF was defined as the need for increased HF therapy in the appropriate clinical setting or HF hospitalization (18). While the secondary endpoint was all-cause mortality. All-cause mortality included death from any cause verified by medical records or telephonic contact. Clinical outcomes were adjudicated by an independent committee to ensure consistency and minimize bias.

### Statistical analysis

For statistical assessment, we utilized SPSS version 26.0 and the R programming language version 4.3.2. Categorical variables were compared between groups using the *χ*^2^ test and expressed as numbers (%). Continuous variables were first tested for normality by Kolmogorov–Smirnov, continuous variables with normal distribution were analyzed by *t*-test, and expressed as mean ± standard deviation, and continuous variables with non-normal distribution were analyzed by Mann–Whitney *U* test.

The cumulative incidence of HF and all-cause mortality during follow-up was estimated using the Kaplan–Meier method, and differences between the male and female groups were compared via the log-rank test. Univariate Cox proportional hazards regression analysis was initially performed to identify potential predictors of HF. Variables with a *P* < 0.05 in univariate analysis or those clinically relevant were subsequently entered into a multivariable Cox regression model to determine the independent association between sex and HF development. Results are presented as hazard ratios (HR) with 95% confidence intervals (CI).

To further evaluate the consistency of the association between sex and HF across different clinical settings, subgroup analyses were conducted according to age, diabetes mellitus, LVEF at hospital discharge, Killip class, and symptoms onset to first medical contact time. Interaction analyses were performed using the likelihood ratio test to assess whether the effect of sex on HF was modified by these baseline characteristics. With a two-sided *P* < 0.05, the difference was considered to be statistically significant.

To further explore the underlying mechanism of the association between sex and new-onset HF, causal mediation analyses were performed based on the counterfactual framework using the R ‘mediation’ package. Non-parametric bootstrapping with 1,000 replications was applied to estimate the mediated effect. The presence of a mediating effect was defined as satisfying all of the following conditions having a significant indirect effect, a significant total effect, and a positive proportion of the mediator effect.

## Results

### Characteristics of the study population

**Figure 1:**
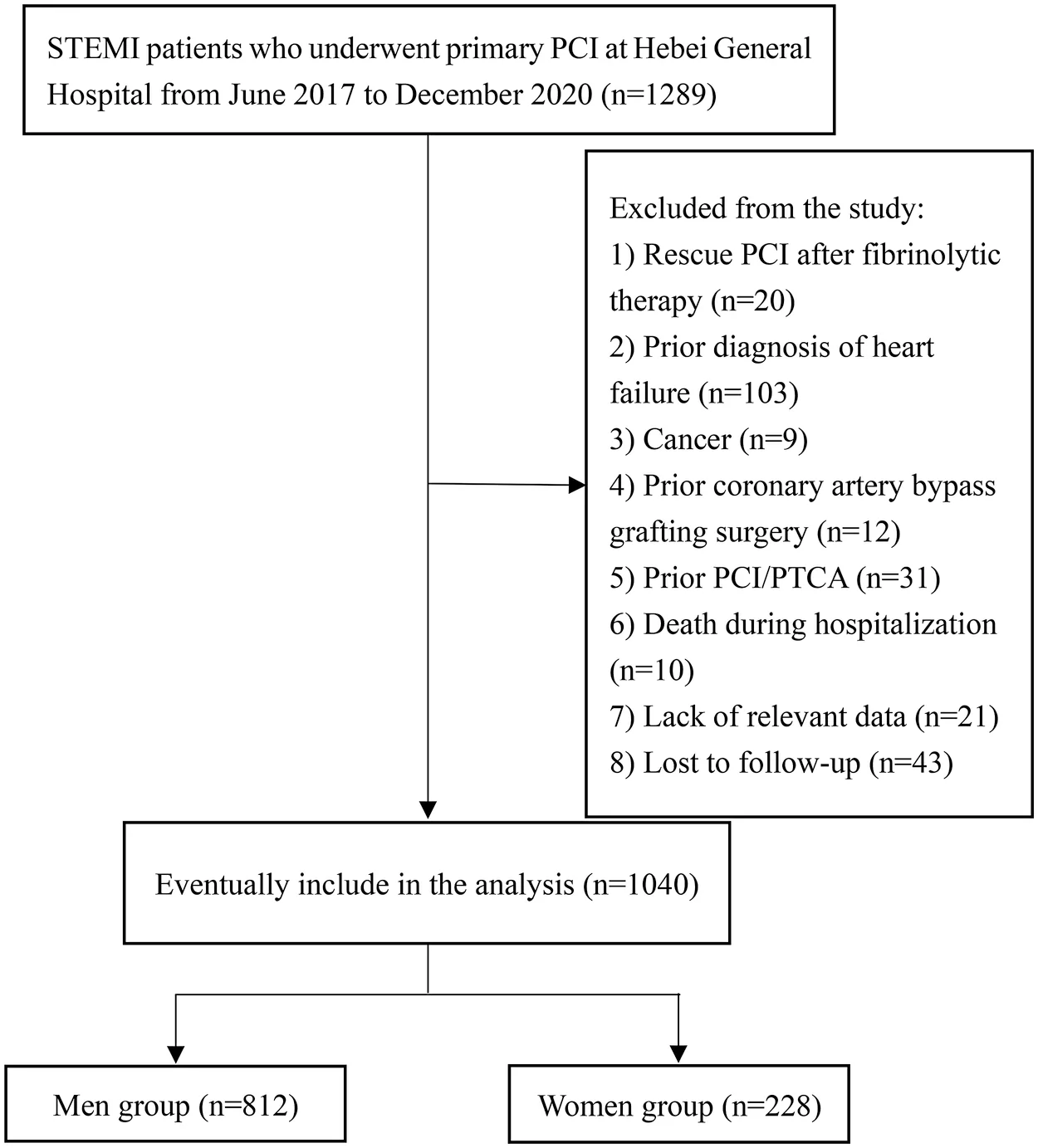
Flow chart of this study. This figure shows the selection criteria and exclusion criteria of the study subjects.

Finally, a total of 1,040 STEMI patients were included in this retrospective study, consisting of 812 (78.1%) men and 228 (21.9%) women. Figure 1 is the flow chart of this study. Baseline characteristics are listed in Table 1. The baseline data of the two groups were compared. There were no significant differences in BMI, hyperlipidemia, LVEF at hospital discharge, peak CK-MB, peak NT-pro BNP, culprit vessel, initial TIMI flow, angiographic no-reflow, number of stents and medication at hospital discharge. Age, hypertension, diabetes mellitus, Killip class, GRACE scores, Glu, peak cTnT, total cholesterol, triglyceride, multivessel disease, high-density lipoprotein cholesterol, low-density lipoprotein cholesterol, door-to-balloon time, heart failure, and all-cause mortality levels were significantly higher in the Women group than in the Men group. However, family history of coronary heart disease, smoking, creatinine, eGFR, uric acid, thrombus aspiration, and symptoms onset to first medical contact <6 h levels were significantly higher in the men group than in the women group.

**Table 1:** Sex differences in baseline characteristics.

Variable	Total (*n* = 1040)	Men (*n* = 812)	Women (*n* = 228)	*P*
Baseline characteristics				
Age, years	62.15 ± 10.26	60.82 ± 9.77	66.91 ± 10.56	<0.001
BMI, kg/m^2^	25.47 (23.65, 27.68)	25.61 (23.88, 27.68)	25.30 (23.23, 27.34)	0.051
Family history of coronary heart disease, *n* (%)	143 (13.75)	123 (15.15)	20 (8.77)	0.014
Smoking, *n* (%)	610 (58.65)	544 (67.00)	66 (28.95)	<0.001
Hypertension, *n* (%)	551 (52.98)	411 (50.62)	140 (61.40)	0.004
Hyperlipidemia, *n* (%)	130 (12.50)	95 (11.70)	35 (15.35)	0.141
Diabetes mellitus, *n* (%)	257 (24.71)	171 (21.06)	86 (37.72)	<0.001
LVEF at hospital discharge, *n* (%)	52.07 ± 8.37	51.96 ± 8.51	52.44 ± 7.86	0.441
Killip class, *n* (%)				<0.001
I	849 (81.63)	686 (84.48)	163 (71.49)	
II	138 (13.27)	90 (11.08)	48 (21.05)	
III	19 (1.83)	11 (1.35)	8 (3.51)	
Ⅳ	34 (3.27)	25 (3.08)	9 (3.95)	
Killip class ≥2, *n* (%)	191 (18.37)	126 (15.52)	65 (28.51)	<0.001
GRACE score	136.00 (115.00, 158.00)	133.00 (111.00, 153.00)	150.50 (132.00, 167.00)	<0.001
Laboratory data				
Glu, mmol/L	6.17 (5.12, 8.46)	6.02 (5.04, 7.90)	7.04 (5.60, 10.41)	<0.001
Peak cTnT, ng/mL	3.34 (2.34, 5.89)	3.43 (2.23, 5.65)	3.71 (3.20, 6.07)	<0.001
Peak CK-MB, U/L	109.50 (53.48, 226.05)	111.30 (49.00, 226.05)	108.35 (63.90, 210.55)	0.727
Peak NT-pro BNP, pg/mL	604.85 (236.00, 1496.25)	597.00 (254.00, 1455.00)	624.00 (236.00, 1700.00)	0.613
Total cholesterol, mg/dL	4.53 (3.90, 5.25)	4.44 (3.87, 5.15)	4.86 (4.13, 5.55)	<0.001
Triglyceride, mg/dL	1.51 (1.05, 2.05)	1.48 (1.03, 2.03)	1.60 (1.11, 2.11)	0.040
High-density lipoprotein cholesterol, mg/dL	1.04 ± 0.26	1.02 ± 0.27	1.09 ± 0.22	<0.001
Low-density lipoprotein cholesterol, mg/dL	2.99 (2.55, 3.48)	2.95 (2.51, 3.42)	3.17 (2.74, 3.67)	<0.001
Creatinine, mg/dL	77.00 (67.60, 87.50)	78.10 (69.21, 88.00)	72.51 (63.45, 86.65)	<0.001
eGFR, mL/min/1.73m^2^	89.75 (76.00, 99.40)	92.40 (80.06, 101.15)	79.41 (63.46, 90.15)	<0.001
Uric acid, mg/dL	330.73 (272.50, 399.05)	338.88 (283.27, 407.62)	294.85 (232.74, 361.20)	<0.001
Procedural data				
Culprit vessel				0.662
Left circumflex artery, *n* (%)	99 (9.52)	79 (9.73)	20 (8.77)	
Left anterior descending artery, *n* (%)	524 (50.38)	409 (50.37)	115 (50.44)	
Left main coronary artery, *n* (%)	62 (5.96)	47 (5.79)	15 (6.58)	
Right coronary artery, *n* (%)	376 (36.15)	286 (35.22)	90 (39.47)	
Multivessel disease, *n* (%)	403 (38.75)	291 (35.84)	112 (49.12)	<0.001
Thrombus aspiration, *n* (%)	304 (29.23)	253 (31.16)	51 (22.37)	0.010
Initial TIMI flow <3, *n* (%)	866 (83.27)	671 (82.64)	195 (85.53)	0.301
Angiographic no-reflow	144 (13.85)	110 (13.55)	34 (14.91)	0.598
Number of stents, *n*	1.18 ± 0.53	1.17 ± 0.53	1.21 ± 0.53	0.266
Symptoms onset to first medical contact <6 h, *n*	883 (84.90)	706 (86.95)	177 (77.63)	<0.001
Door-to-balloon time, min	60.00 (47.00, 79.00)	60.00 (47.00, 79.00)	66.50 (53.00, 85.00)	0.006
Medication at hospital discharge, *n* (%)				
Statin	919 (88.37)	716 (88.18)	203 (89.04)	0.721
Beta-blocker	826 (79.42)	649 (79.93)	177 (77.63)	0.449
Calcium channel blocker	257 (24.71)	209 (25.74)	48 (21.05)	0.147
ACEI or ARB	541 (52.02)	423 (52.09)	118 (51.75)	0.928
Heart failure, *n* (%)	204 (19.62)	145 (17.86)	59 (25.88)	0.007
All-cause mortality, *n* (%)	82 (7.88)	53 (6.53)	29 (12.72)	0.002

BMI, body mass index; LVEF, left ventricular ejection fraction; GRACE, global registry of acute coronary events; Glu, fasting blood glucose; NT-pro BNP, N-terminal pro brain natriuretic peptide; TIMI, thrombolysis in myocardial infarction; CK-MB, creatine kinase isozyme; cTnT, cardiac troponin T; ACEI, angiotensin-converting enzyme inhibitors; ARB, angiotensin receptor blockers; eGFR, estimated glomerular filtration rate.

### Regression analysis of risk factors for heart failure

**Figure 2:**
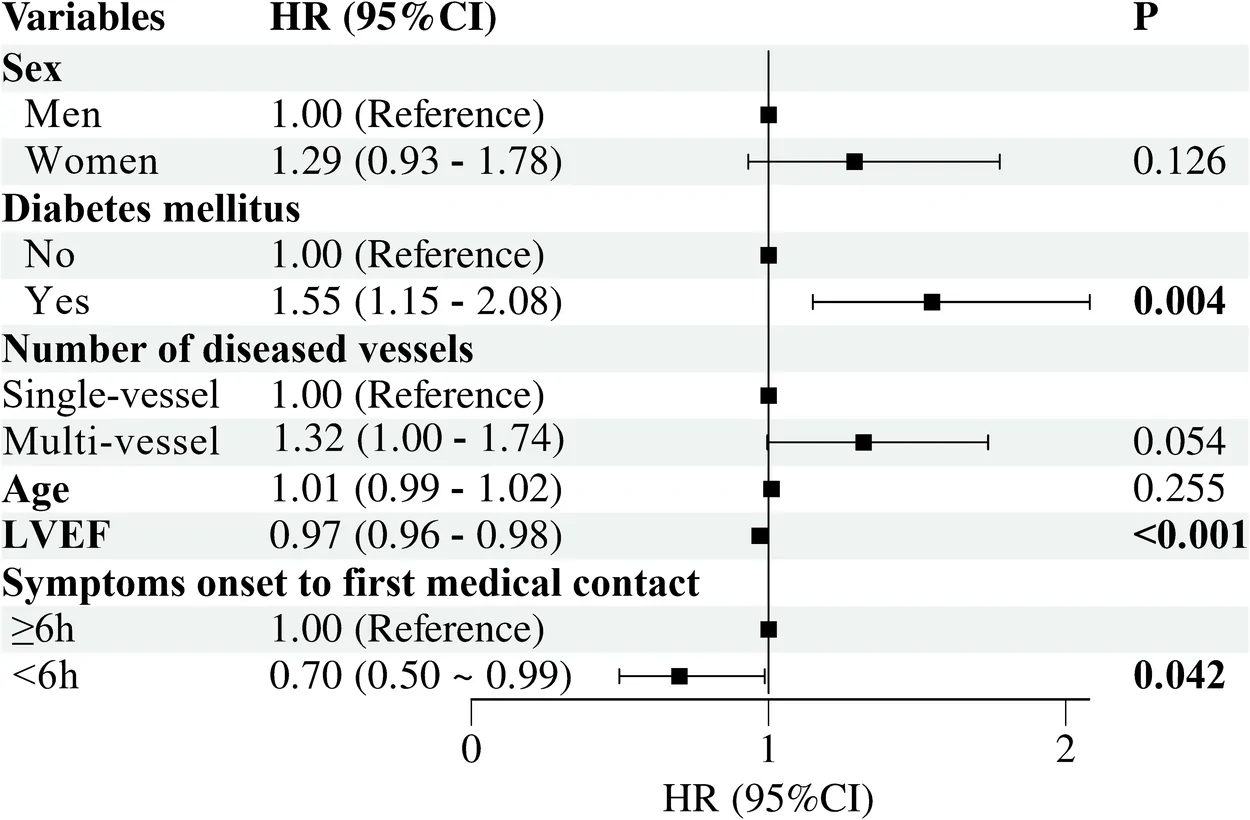
Forest plot of multivariate analysis assessing the association of different variables with the development of heart failure.

In the univariate Cox proportional hazards analysis, several baseline factors were significantly associated with an increased risk of HF, including women, advanced age, diabetes mellitus, and multivessel disease. Conversely, higher LVEF and symptoms onset to first medical contact time were associated with a lower risk of post-STEMI HF. Baseline variables including hypertension, serum creatinine, and eGFR did not achieve statistical significance in the univariate Cox regression analysis (all *P* > 0.05) and were consequently excluded from the final multivariable model in accordance with our pre-specified statistical protocol. After adjusting for age, sex, and procedural factors, diabetes mellitus, LVEF at hospital discharge, and symptom onset to first medical contact < 6 h remained as independent predictors of HF (Table 2; Figure 2).

**Table 2:** Effects of various variables on HF in univariate and multivariate regression analyses.

Variables	Univariate analysis		Multivariate analysis	
	HR (95% CI)	*P*	HR (95% CI)	*P*
Women	1.51 (1.12–2.05)	0.007	1.29 (0.93–1.78)	0.126
Age	1.02 (1.01–1.03)	0.028	1.01 (0.99–1.02)	0.255
Diabetes mellitus	1.59 (1.19–2.13)	0.002	1.55 (1.15–2.08)	0.004
LVEF	0.97 (0.96–0.99)	< 0.001	0.97 (0.96–0.98)	<0.001
Multivessel disease	1.33 (1.01–1.75)	0.043	1.32 (1.00–1.74)	0.054
Symptoms onset to first medical contact <6h	0.66 (0.47–0.92)	0.015	0.70 (0.50–0.99)	0.042

### Incidence of heart failure and all-cause mortality

**Figure 3:**
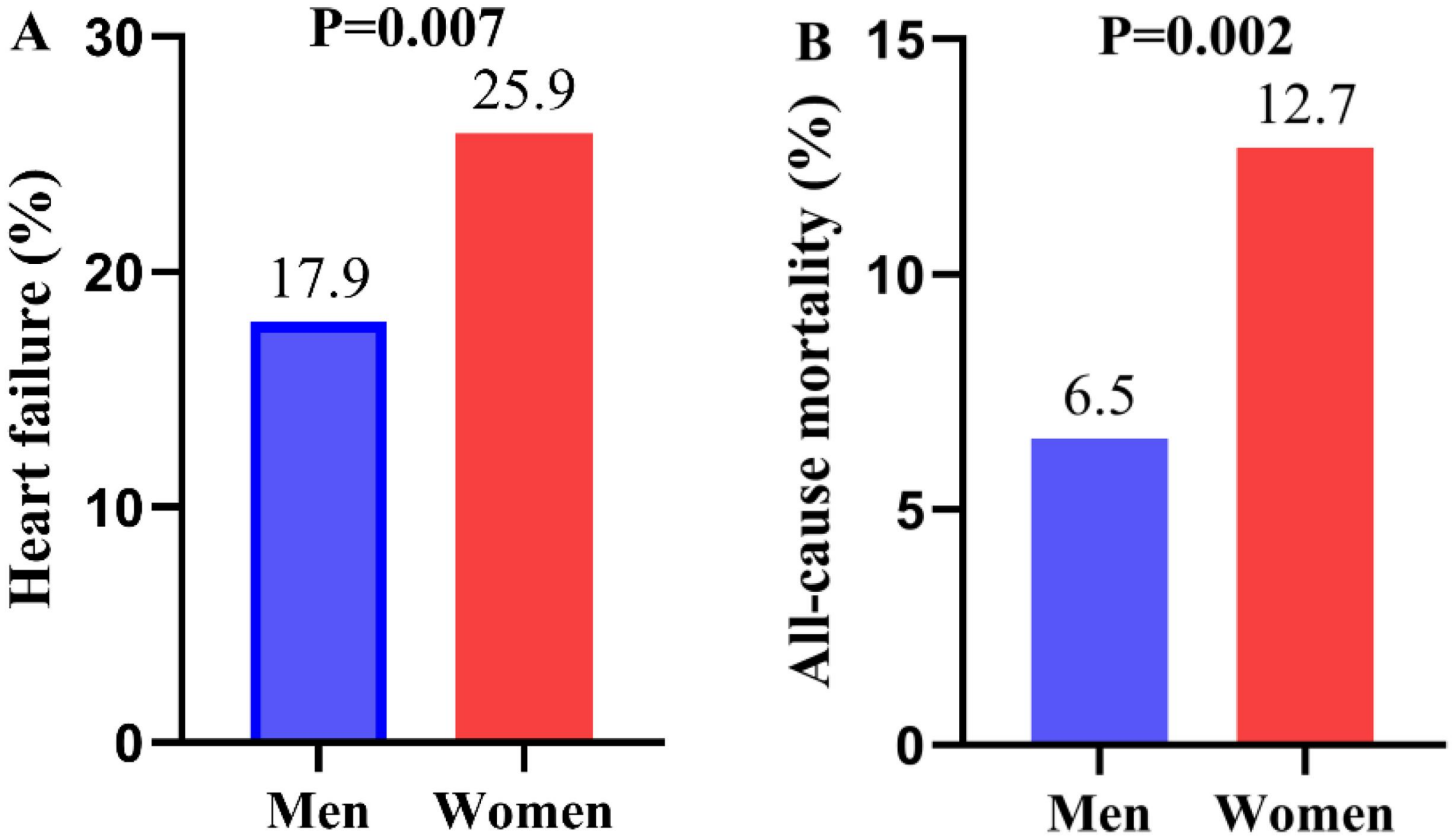
**(A)** Sex differences in HF in the cohort during follow-up. **(B)** Sex difference in all-cause mortality during follow-up.

**Figure 4:**
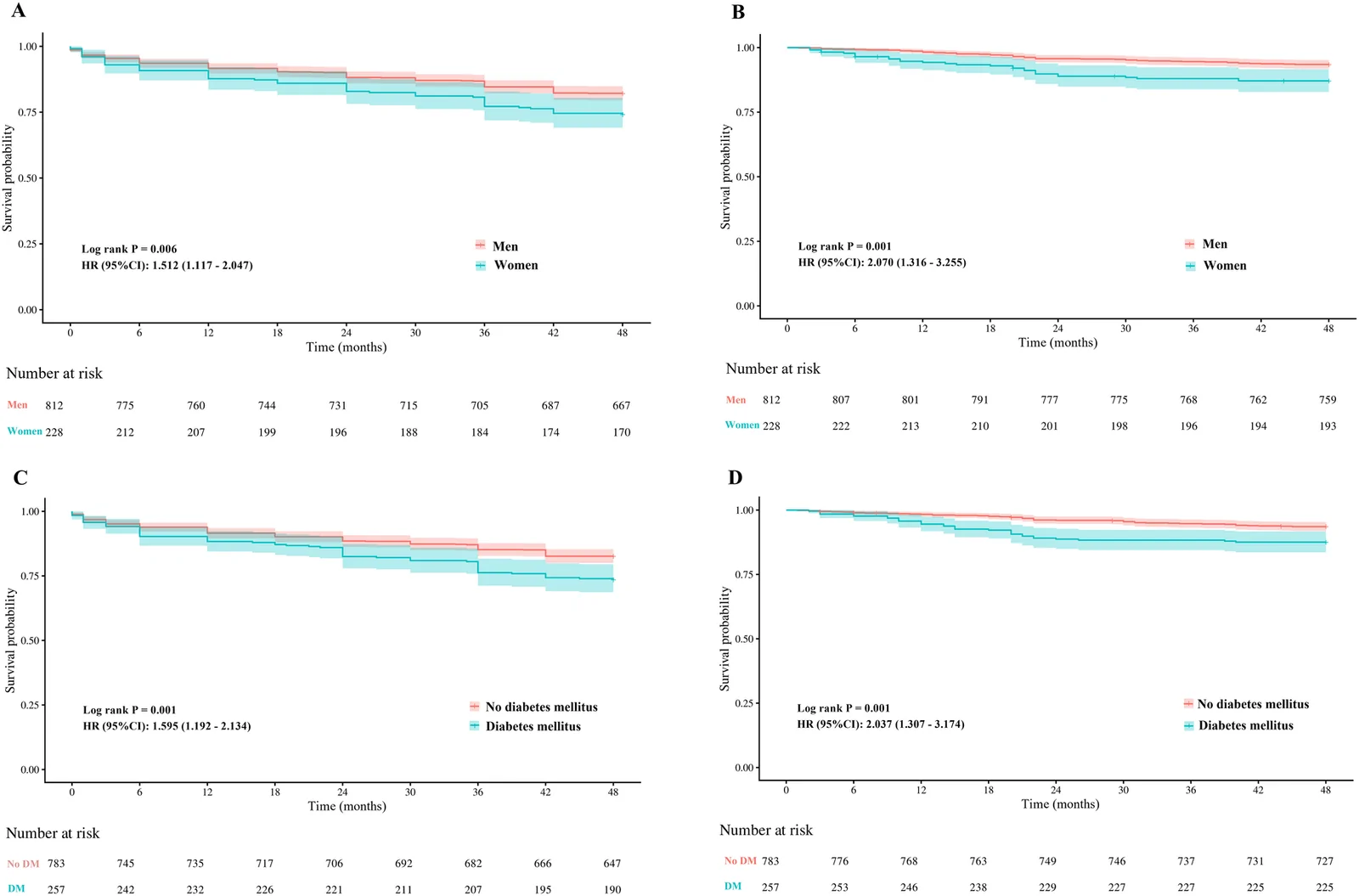
Kaplan–Meier cumulative event curves, according to sex and diabetes mellitus status. Time to cumulative event rate in sex with STEMI according to the presence of HF **(A)** and all-cause mortality **(B)** during the follow-up. Time to cumulative event rate in diabetes mellitus status with STEMI according to the presence of HF **(C)** and all-cause mortality **(D)** during the follow-up.

During follow-up, the incidence of HF was significantly higher in women than in men. Similarly, all-cause mortality was markedly elevated in the female cohort (Figure 3). The Kaplan–Meier analysis confirmed that the cumulative incidence rates of HF and all-cause mortality in female patients with STEMI were significantly higher than those in male patients. The cumulative incidence of HF and all-cause mortality in STEMI patients with diabetes mellitus was significantly higher than that in patients without diabetes mellitus (Figure 4).

### Subgroup and interaction analysis

**Figure 5:**
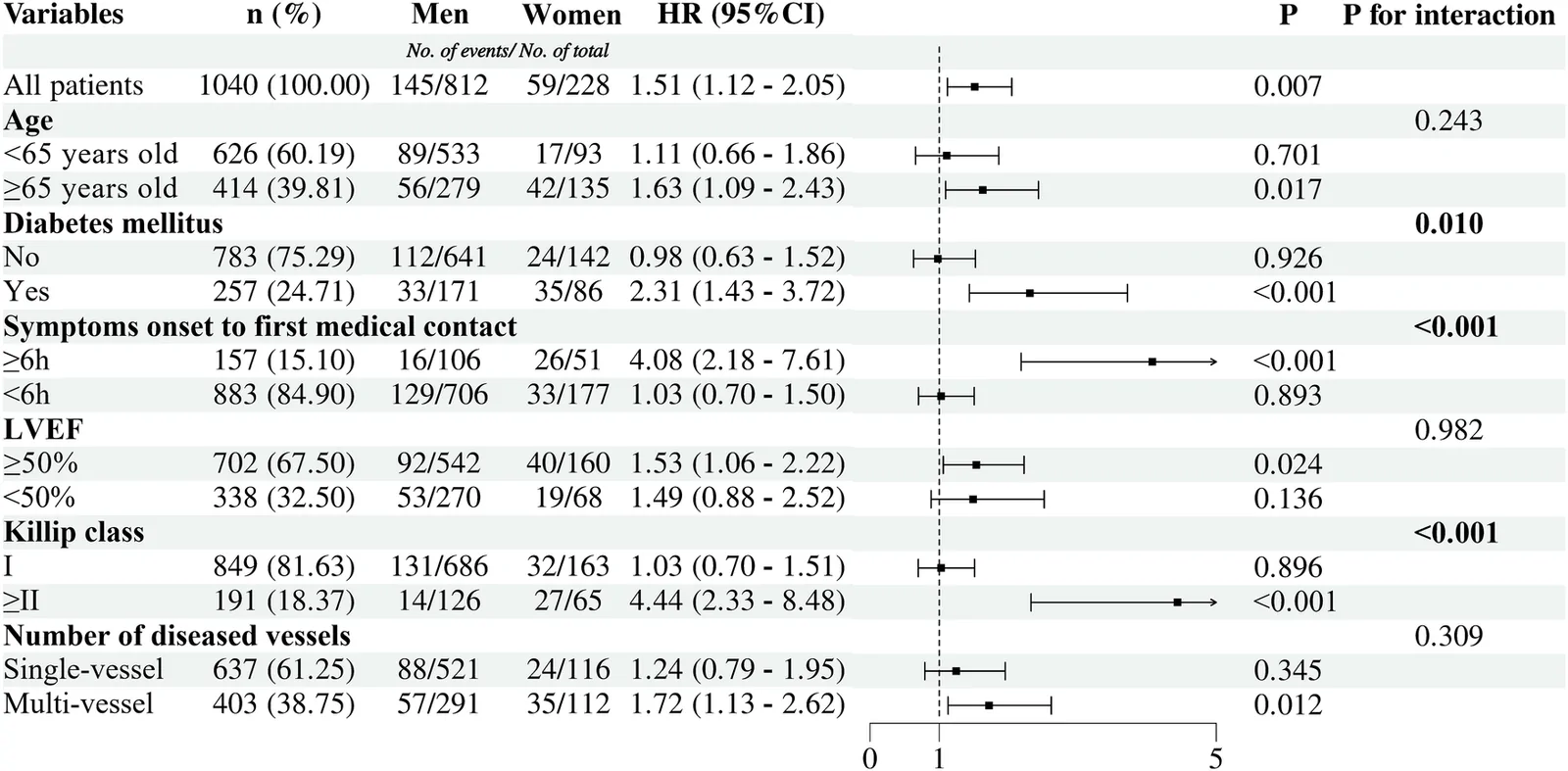
Forest plot of multivariate analysis assessing the association of different variables with the development of heart failure.

The subgroup analysis results showed that there was a significant interaction between sex and diabetes mellitus status, Killip class, and symptoms onset to first medical contact time (Table 3; Figure 5).

**Table 3:** Subgroup and interaction analyses.

Subgroup	Level	Men (Events/N)	Women (Events/N)	HR (95% CI)	*P* for Interaction
Age	<65 vs. ≥65	56/279	42/135	1.63 (1.09–2.43)	0.243
Diabetes mellitus	Yes vs. No	33/171	35/86	2.31 (1.43–3.72)	0.010
LVEF at hospital discharge	＜50% vs. ≥50%	53/270	19/68	1.49 (0.88–2.52)	0.982
Multivessel disease	Single vs. Multi	57/291	35/112	1.72 (1.13–2.62)	0.309
Killip class	I vs. ≥ II	14/126	27/65	4.44 (2.33–8.48)	<0.001
Symptoms onset to first medical contact <6h	<6 h vs. ≥6h	129/706	33/177	1.03 (0.70–1.50)	<0.001

### Causal mediation analysis

**Figure 6:**
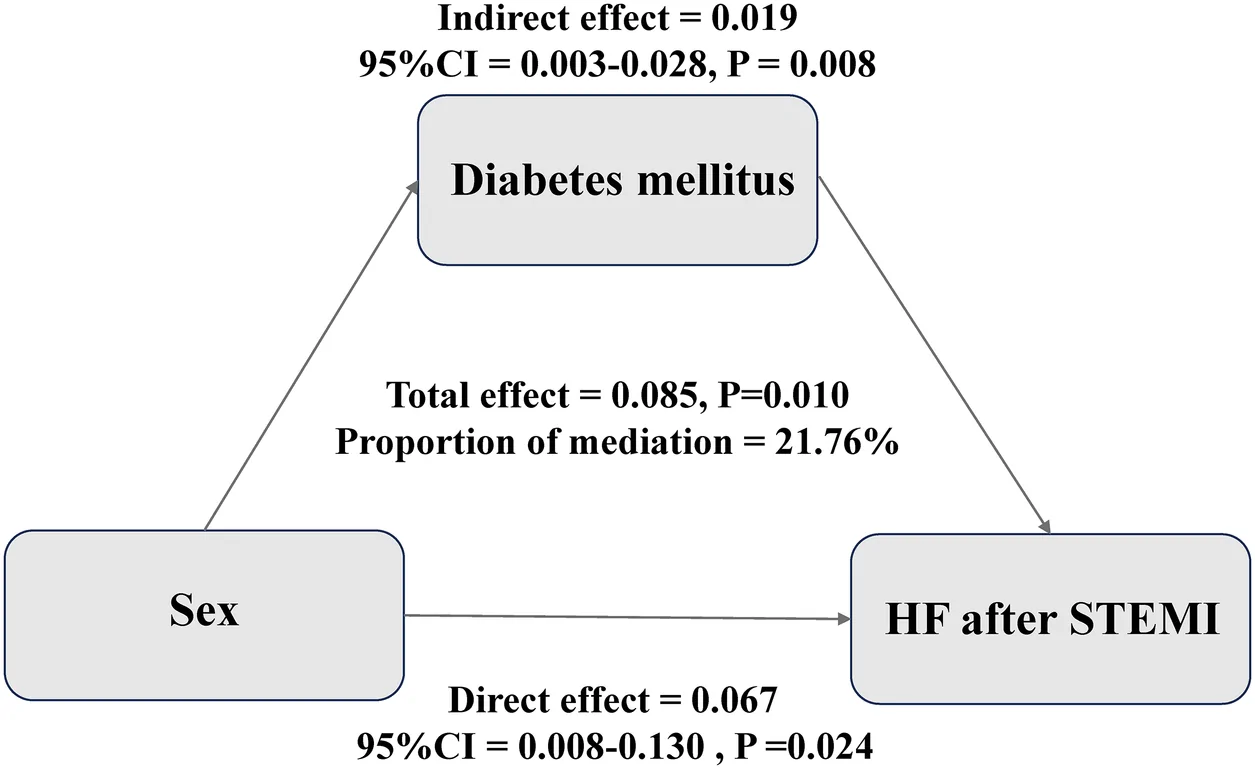
Analysis of the mediation by diabetes mellitus of the associations of sex with HF after STEMI.

To clarify whether the impact of sex on post-STEMI HF was mediated by key clinical characteristics, we conducted causal mediation analyses for diabetes mellitus, age, and door-to-balloon time. As shown in Figure 6, diabetes mellitus acted as a significant mediator, explaining 21.76% of the total effect of sex on new-onset HF (Indirect Effect = 0.019, 95% CI: 0.003–0.028, *P* = 0.008). Conversely, no significant mediating effects were observed for age (*P* = 0.108) or door-to-balloon time (*P* = 0.904).

## Discussion

In this contemporary cohort study of 1,040 STEMI patients, we observed that while women exhibited a significantly higher crude incidence of post-infarct heart failure and mortality, sex was not an independent predictor of these adverse outcomes after rigorous multivariable adjustment. Our findings suggest that the observed sex disparities are predominantly driven by the advanced age, greater comorbidity burden, and prolonged ischemic times prevalent among women. Notably, interaction analyses revealed that the deleterious impact of diabetes mellitus, higher Killip class, and delayed medical contact on heart failure risk was significantly amplified in the female population.

Previous studies have shown that HF is a serious complication of STEMI and has an important impact on the clinical prognosis of patients with STEMI (19). The risk of HF in female patients with STEMI is higher than that in male patients (20). In contrast, a myocardial infarction (MI) cohort study by Ezekowitz et al. showed a higher rate of acute heart failure in male patients with a first hospitalization for MI (21). This discrepancy in study results may be related to the differences in the populations included in the studies. This study included only STEMI patients undergoing primary PCI, whereas the study by Ezekowitz et al. included both non-STEMI and STEMI patients. However, the ischemia-reperfusion time and treatment strategy selection are different between non-STEMI and STEMI (22). In recent years, the gender differences in cardiovascular diseases have been a research hotspot. Numerous studies have shown that compared with male patients, female patients are older, have a lower smoking rate, and are more likely to have chronic diseases such as diabetes and hypertension (23–26). Female patients with AMI often exhibit more atypical symptoms, which leads to delays in diagnosis and treatment.

Consistent with previous research results, in this study, female patients had a higher baseline risk, including a higher proportion of those with advanced age, hypertension, and diabetes, as well as a higher GRACE risk score. This is further supported by our mediation analysis, suggesting that aggressive metabolic management in female STEMI patients post-discharge is a critical target to reduce sex-related disparities. Moreover, the rates of post-MI HF and all-cause mortality were significantly higher. The underlying mechanism for these differences in clinical characteristics lies in the following: On the one hand, young women are influenced by the protective effect of estrogen on the heart, and the occurrence of AMI often depends on a greater load of cardiovascular risk factors (27). On the other hand, the withdrawal of estrogen during menopause leads to endothelial dysfunction and the accumulation of lipids in the blood vessels, which significantly increases the risk of AMI and causes women to generally have a later onset age (28).

An important finding in our study was that female patients exhibited significantly longer door-to-balloon times. Delayed reperfusion is a well-established driver of expanded infarct size and subsequent heart failure. This clinical lag in women is largely attributed to sex-specific symptom profiles; as highlighted by van Oosterhout et al., the high prevalence of non-chest pain symptoms in female patients frequently misleads initial clinical triage, creating substantial institutional delays before the coronary intervention is initiated (10). Our interaction analysis reinforces this mechanism, demonstrating that a time from symptom onset to reperfusion exceeding 6 h disproportionately penalties female outcomes, culminating in a more than fourfold increased risk of heart failure. Our data highlight that efforts to reduce prehospital delays remain key to improving woman-specific outcomes in STEMI. In addition, the significant interaction between sex and diabetes requires clinical attention. We found that women with diabetes had a significantly higher risk of developing heart failure than men with diabetes. This “double whammy” of female sex and impaired glucose metabolism may involve sex-specific microvascular dysfunction as well as more severe diffuse coronary artery disease (29). To further clarify whether sex disparities were driven by overall clinical presentation rather than biological sex alone, extended phenotypical clustering evaluations indicated that within both low- and moderate-risk clinical clusters, the incidence of post-STEMI heart failure did not significantly differ between women and men. Excess risk in female patients was exclusively concentrated in the ultra-high-risk cohort with severe comorbidity burden, reinforcing that baseline age and metabolic profile are the predominant drivers of the observed crude risk differences.

Intriguingly, after controlling for baseline clinical profiles, biological sex was no longer an independent predictor of post-STEMI heart failure in our cohort, indicating that the observed disparity is primarily comorbidity-driven rather than an isolated biological determinism. This finding aligns with the conceptual framework proposed by Ferraz-Torres, whose recent mixed-methods study illustrated that gender-related disparities and management gaps heavily bias the prognostic trajectory of patients with acute coronary syndrome. In high-risk clinical archetypes, such as post-menopausal females, the biological burden of aggressive comorbidities (e.g., type 2 diabetes mellitus) intertwines with systemic management nuances (30). This is strongly corroborated by our causal mediation analysis, wherein diabetes mellitus acted as a powerful mediator, capturing 21.76% of the total effect of sex on new-onset heart failure.

### Limitations

Our study still has limitations. Firstly, as this is a single-center retrospective study, there may be potential biases. Secondly, we were unable to obtain the patients’ hormone and related inflammatory biomarker data, thus failing to further elucidate the mechanisms behind these gender differences. Thirdly, due to the retrospective nature of this study, serial longitudinal data on LVEF and NT-pro BNP during the follow-up period were not systematically captured for all patients, which prevented us from tracking the dynamic progression of cardiac function. Fourthly, we could not definitively classify the new-onset heart failure events into specific phenotypes, such as heart failure with reduced (HFrEF) vs. preserved ejection fraction (HFpEF). Given that women presented with an advanced age and higher comorbidity burden, a substantial portion of their post-STEMI heart failure might be driven by an underlying predisposition to HFpEF independent of the index myocardial infarction.

## Conclusion

In this cohort study of STEMI patients, women experienced a higher crude incidence of HF and mortality than men. However, female was not an independent predictor of HF after adjusting for baseline comorbidities and reperfusion delays. The observed sex disparities were primarily driven by advanced age and a higher prevalence of diabetes mellitus in women. Notably, diabetes and higher Killip class disproportionately increased HF risk in the female population, suggesting that sex-specific risk stratification and timely intervention are essential to improve outcomes in high-risk women.

## Data Availability

The datasets used and/or analyzed during the current study are available from the corresponding author on reasonable request.
